# Organ-specific and tumor-size-dependent responses to sunitinib in clear cell renal cell carcinoma

**DOI:** 10.1186/1471-2490-14-26

**Published:** 2014-03-11

**Authors:** Norihiko Tsuchiya, Takeshi Yuasa, Shinya Maita, Shintaro Narita, Takamitsu Inoue, Kazuyuki Numakura, Mitsuru Saito, Shigeru Satoh, Junji Yonese, Tomonori Habuchi

**Affiliations:** 1 Department of Urology, Akita University Graduate School of Medicine, Akita, Japan; 2 Department of Urology, Cancer Institute Hospital, Japanese Foundation for Cancer Research, Tokyo, Japan

**Keywords:** Advanced renal cell carcinoma, Sunitinib, Tumor size, Tumor response, C-reactive protein

## Abstract

**Background:**

Tyrosine kinase inhibitors (TKIs) have been used as standard therapy for patients with advanced renal cell carcinoma (RCC). However, information on factors predicting response to treatment with TKIs is lacking. This study aimed to assess the association between initial tumor size, involved organs, pre-treatment C-reactive protein (CRP) levels, and reduction in tumor size in patients with clear cell RCC (CCRCC) treated with sunitinib.

**Methods:**

Patients with advanced CCRCC with target lesions with a maximum diameter ≥ 10 mm treated with sunitinib were evaluated. The tumor diameter representing the best overall response was designated as the post-treatment tumor diameter.

**Results:**

A total of 179 lesions in 38 patients were analyzed. Organ-specific analysis demonstrated that pre-treatment diameter of lung metastatic lesions had a moderate inverse association with percent reduction in post-treatment tumor diameter (R = 0.341). Lung lesions showed significantly greater percent reductions in diameter than liver and kidney lesions (*P* = 0.007 and 0.002, respectively). Furthermore, based on a CRP cut-off level of 2.0 mg/dl, mean tumor size reduction was significantly greater in patients with low CRP levels than in patients with high CRP levels in lesions with diameters < 20 mm (*P* = 0.002). CRP level had no effect on mean size reduction in lesions with a diameter ≥ 20 mm.

**Conclusions:**

Patients with CCRCC with smaller lung metastatic lesions and lower CRP levels may achieve greater percent reductions in tumor size with sunitinib therapy than patients with extra-pulmonary lesions, large lung lesions, and/or higher CRP levels.

## Background

In the era of cytokine therapy, tumor response to treatment in advanced or metastatic renal cell carcinoma (RCC) has been reported to vary according to the organs involved [1,2]. Longer overall survival and a higher response rate to therapy with interferon-α or a combination of interleukin-2 and interferon-α were observed in patients with only lung metastasis, compared with those with extra-pulmonary metastasis [1,2]. Complete remission (CR) after treatment with tyrosine kinase inhibitors (TKIs), which mainly target vascular endothelial growth factor receptors, remains a rare event, but most patients who do achieve CR have either lung metastasis alone, or only lymph node involvement [3,4]. However, most cancer clinical trials evaluate tumor response using the response evaluation criteria in solid tumors (RECIST), in which the longest diameters of target lesions in multiple organs are summed. Tumor response in individual metastatic lesions in specific organs has not been delineated.

A reduction in tumor size >10%, calculated as the sum of the longest diameter of the target lesions, was significantly associated with both time to treatment failure and overall survival, suggesting that size reduction of target lesions may predict the outcome of treatment with TKIs [5]. In addition, Yuasa et al. recently demonstrated that a smaller initial tumor size predicted a good response to TKIs, and that the maximum response was achieved in lung lesions [6]. TKIs have shown significant clinical benefit in advanced clear cell RCC (CCRCC) in large randomized trials [7-9]. However, the reported objective responses vary according to the different types of TKIs, and a recent phase II trial failed to demonstrate any clinical efficacy of sunitinib in non-CCRCC [10]. Tumor size reduction may thus be affected by many factors, including initial tumor size, involved organs, tumor histology, tumor aggressiveness, or type of TKI used. In this study, we evaluated the association between initial tumor size of individual lesions in specific organs and reduction in tumor size in patients with CCRCC treated with sunitinib.

## Methods

### Patients and tumor measurement

A total of 38 patients with advanced CCRCC, who received at least two cycles of sunitinib at Akita University Hospital and at the Cancer Institute Hospital of the Japanese Foundation for Cancer Research, were enrolled in this institutional, review-board-approved, retrospective study. Pathological diagnosis was made by radical nephrectomy in 30 patients and by percutaneous biopsy in eight patients who were not indicated for surgical treatment, because of a significantly higher total volume of metastatic lesions compared with the primary lesion. The initial dose of sunitinib was 50 mg/day, which was reduced to 37.5 mg/day based on the patient’s physique, age, and performance status. Sunitinib was initiated on a 28 days on/14 days off schedule, and a dose reduction to 25 mg/day or complete cessation was considered in the event of grade 3 or higher toxicity, according to the Common Terminology Criteria for Adverse Events (CTC-AE). All lesions were evaluated using a multidetector computed tomography scanner, and lesions ≥ 10 mm in diameter were considered target lesions. The maximum diameter of each target lesion was measured before treatment with sunitinib (pre-treatment tumor diameter) and every 2–3 months thereafter. The tumor diameter at the point when best overall response was achieved, based on the RECIST version 1.0, was adopted as the post-treatment tumor diameter. In this study, the most common metastatic organs, including lung, liver, and lymph nodes, as well as the kidney, were subjected to analysis.

### Statistical analysis

The association between pre-treatment tumor diameter and percent change between pre- and post-treatment tumor diameters for each lesion was assessed by Pearson’s correlation coefficient. The Kruskal Wallis test was used to compare differences in percent change in tumor diameter between the four different organs. The Mann–Whitney U test was used to compare differences between two groups. A receiver-operator curve (ROC) was constructed to find the pre-treatment tumor diameter predicting tumor response to sunitinib treatment. A value of *P* < 0.05 was considered statistically significant.

## Results

### Patients and target lesions

The patients included 30 men and eight women with a median age of 62 years (range 27–81 years). The patients’ characteristics are listed in Table 1. The best response to sunitinib treatment was CR in one patient (3%), partial response (PR) in 11 (29%), stable disease (SD) in 23 (61%), and progressive disease (PD) in three (8%). The objective response rate was 32% and the clinical benefit rate (CR + PR + SD for at least 3 months) was 92%. A total of 179 lesions ranging from 10 to 106 mm were measured and analyzed in 38 patients. These lesions were localized as follows: 124 in the lung, 12 in the liver, 24 in the lymph nodes, and 19 in the kidney. Of the 15 patients with kidney tumors, seven who underwent nephrectomy had target lesions in the contralateral kidney, including two patients with multiple lesions. The remaining eight patients had primary kidney tumors that were diagnosed by percutaneous needle biopsy.

**Table 1:** Patients’ characteristics

**Characteristic**	**No. of patients (%)**
**Sex**	
Male	30 (78.9)
Female	8 (21.1)
**Age, y**	
Median [range]	62 [27–81]
**ECOG performance status**	
0	25 (65.8)
1	7 (18.4)
> 1	6 (15.8)
**MSKCC risk category**	
Favorable	8 (21.1)
Intermediate	20 (52.6)
Poor	10 (26.3)
**Target organs**	
Lung	31 (81.6)
Liver	6 (15.8)
Lymph node	11 (28.9)
Kidney	15 (39.5)
**Nephrectomy**	
Yes	30 (78.9)
No (biopsy)	8 (21.1)
**Prior treatments**	
None	27 (71.1)
Cytokines alone	4 (10.5)
Sorafenib ± cytokines	7 (18.4)

### Associations between pre-treatment tumor diameter and percent change in target lesion size in different organs

**Figure 1:**
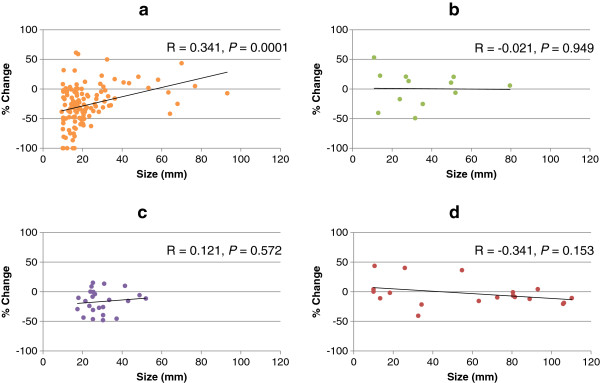
**Association between pre-treatment tumor size and percent change in lesion size after sunitinib treatment.** Association between pre-treatment tumor size and percent change in size was analyzed for lesions in the lung **(a)**, liver **(b)**, lymph nodes **(c)**, and kidney **(d)**. The pre-treatment size of lung lesions showed a moderately positive association with percent size change after treatment with sunitinib, while no association was observed in the liver, lymph nodes, and kidney.

**Figure 2:**
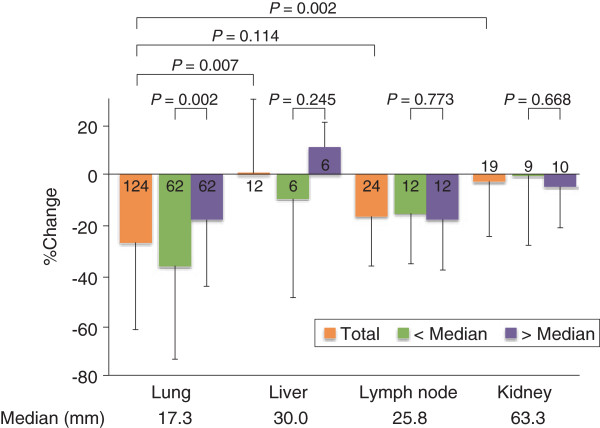
**Percent change in target lesion size in different organs.** The reduction in lesion size was significantly greater in lung lesions compared with liver and kidney lesions. Lung lesions with an initial diameter < 17.3 mm (median) showed a significant percent reduction in size compared with lesions ≥ 17.3 mm. No significant differences in relation to initial lesion size were observed in the liver, lymph nodes, and kidney.

The associations between pre-treatment tumor diameter and percent change in size of each target lesion in each of four organs were analyzed separately. Organ-specific analysis demonstrated that pre-treatment diameter of lung metastatic lesions had a moderately positive association with percent change in post-treatment tumor diameter (R = 0.341, Figure 1). There were fewer target lesions in the other three organs than in the lung, and there was no association between liver, lymph node, or kidney lesion size and percent change in post-treatment tumor diameter (Figure 1). The percent changes in target lesion size differed significantly between the four individual organs (*P* = 0.0007 by the Kruskal Wallis test). The mean (± SD) percent changes in target lesion size in the lung, liver, lymph nodes, and kidney were −27.1 ± 33.5, 0.5 ± 29.4, −16.7 ± 19.6, and −2.7 ± 21.7, respectively. Target lesions in the lung showed the greatest change in size, which was significantly greater than in the liver and kidney (*P* = 0.007 and 0.002, respectively). There was no difference in the percent change in target lesion size between the lung and lymph nodes (*P* = 0.114) (Figure 2).

Lesions in each organ were divided into two groups according to the median pre-treatment diameter, and percent changes in tumor diameter were compared between the two groups. Lung lesions with a pre-treatment diameter less than the median value of 17.3 mm showed a significant percent reduction in diameter compared with tumors ≥ 17.3 mm (*P* = 0.002). There were no differences in the percent change in relation to size above or below the median value in other organs (Figure 2).

### Cut-off value for pre-treatment tumor diameter predicting response to sunitinib in lung metastasis

**Figure 3:**
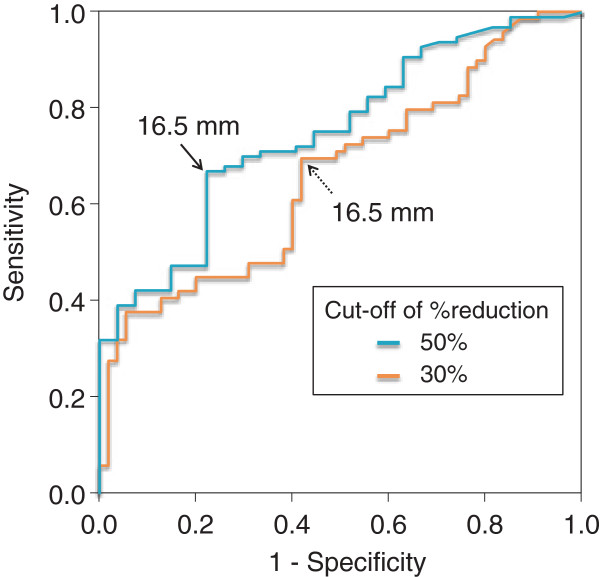
**Cut-off values of initial lung tumor size for predicting 30% and 50% reductions in diameter after sunitinib treatment.** Based on ROC analysis, the cut-off values of initial lung lesion size for predicting reductions in diameter of both 30% and 50% were 16.5 mm.

ROC curves were drawn to determine cut-off values predicting 30% and 50% reductions in the diameter of lung metastatic lesions (Figure 3). The cut-off predicting a 30% reduction in diameter was 16.5 mm, with a sensitivity of 69.6%, specificity of 58.2%, and an area under the curve (AUC) of 0.662. The cut-off predicting a 50% reduction in diameter was also 16.5 mm, with a sensitivity of 67.0%, specificity of 77.8%, and an AUC of 0.752. Using this cut-off value, the percent change in lesion size for lesions < 16.5 mm was −41.7 ± 35.5%, while that for lesions ≥ 16.5 mm was −16.2 ± 26.9% (*P* = 0.0005).

### Influence of pre-treatment CRP value, cytoreductive nephrectomy, and treatment line on percent change in target lesion size in the lung

**Figure 4:**
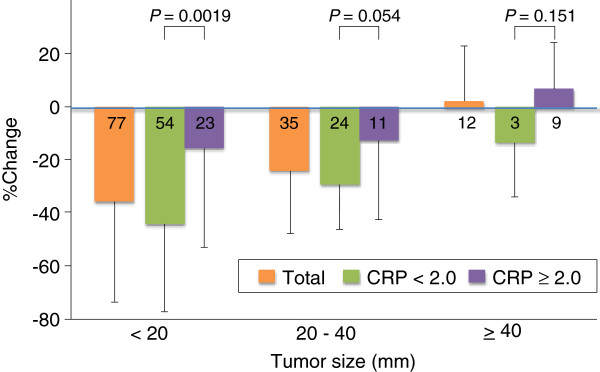
**Influence of CRP on percent change in lung lesion size.** In patients with lung lesions < 20 mm, the percent reduction in size was significantly greater in patients with low, compared with high, CRP levels. Meanwhile, CRP level had a marginal effect on size reduction in lesions ≥ 20 to < 40 mm, and no effect in lesions ≥ 40 mm.

Metastatic lung lesions were categorized into two groups based on pre-treatment C-reactive protein (CRP) levels. The mean diameter and percent change in lesion size in patients with CRP < 2.0 mg/ml were 19.0 ± 9.3 mm and −38.3 ± 30.5%, respectively, while those in patients with CRP ≥ 2.0 mg/ml were 28.3 ± 20.6 mm and −9.6 ± 33.9%, respectively. The lesions were further divided into three subgroups according to pre-treatment diameter < 20 mm, ≥ 20 to < 40 mm, and ≥ 40 mm. Percent changes were compared between lesions in patients with CRP < 2.0 mg/dl (low CRP) and those with CRP ≥ 2.0 mg/dl (high CRP) in each subgroup. For lesions < 20 mm, patients with low CRP had significantly greater reductions in tumor diameter than patients with high CRP (−43.8 ± 33.4% vs. −15.3 ± 37.7%, *P* = 0.0019). In patients with lesions ≥20 to < 40 mm, there was a tendency towards a greater reduction in patients with low CRP compared with high CRP, though the difference was not significant (−29.0 ± 17.3% vs. −12.4 ± 30.2%, *P* = 0.054), and similarly there was no significant association between tumor reduction and CRP level in patients with lesions ≥ 40 mm (−13.3 ± 20.6 vs. 6.8 ± 17.4, *P* = 0.151) (Figure 4).

Percent changes in size were compared between lung lesions in patients in each diameter subgroup who did and did not undergo cytoreductive nephrectomy. There were no lung lesions ≥ 40 mm in patients who did not undergo cytoreductive nephrectomy. There was no significant difference between mean percent change in lesion size in patients with and without cytoreductive nephrectomy (−36.1 ± 32.6 vs. −28.2 ± 41.9, *P* = 0.421 and −20.4 ± 25.1 vs. −32.0 ± 21.9, *P* = 0.307 in lesions < 20 mm and ≥ 20 mm, respectively).

Similarly, there was no significant difference in percent change in lesion size between lung lesions in patients treated as first-line therapy and those treated as second-line or later therapy in any diameter subgroup (−38.1 ± 37.7 vs. −32.1 ± 33.9, *P* = 0.280; −23.5 ± 21.6 vs. −17.5 ± 23.3, *P* = 0.803; and 3.5 ± 2.6 vs. 1.9 ± 22.9, *P* > 0.9 in lesions < 20 mm, ≥ 20 to < 40 mm, and ≥ 40 mm, respectively).

## Discussion

A previous study on metastatic RCC by Yuasa et al. demonstrated that: 1) smaller initial tumor size predicted a good response to TKIs; 2) the greatest response was achieved in patients with lung lesions; and 3) there was no difference in tumor response between patients treated with sorafenib and sunitinib [6]. However, these results raised several specific questions. First, tumor histology and progression risk may affect the response to TKIs. TKIs are associated with a good response in patients with CCRCC, but are less effective against non-CCRCC [10]. Similarly, patients with favorable risk factors have a greater chance of a good tumor response than those with poorer risk factors. Second, there is the possibility of bias in terms of the types of TKI selected; given that sunitinib showed a higher response rate than sorafenib [7,8], patients with larger or more rapidly-growing tumors may be allocated sunitinib rather than sorafenib in clinical practice. Third, efficacy based on initial tumor size may differ between different organs; although the previous study compared mean lesion-size reductions between different organs, they did not compare the effect of initial tumor size in individual organs. It is therefore unclear if the association between initial lesion size and tumor response was observed in each organ, or if the association could be attributed to the fact that most of the small lesions were lung metastases, which showed a good response to TKIs. The current study only included CCRCC patients treated with sunitinib. We found that lung lesions showed the greatest response to sunitinib, and detected a modest correlation between initial tumor diameter and reduction in lesion size, while even small lesions in other organs failed to respond. However, the number of extra-pulmonary tumors assessed was too small to determine statistical significance, and further studies with larger numbers of tumors are needed to obtain conclusive results.

Only lesions with an initial diameter < 20 mm achieved a CR in this study, indicating that a lung-tumor reduction of > 50% might be limited to smaller lesions. The cut-off value of 16.5 mm for a > 50% reduction in diameter was calculated using ROC analysis, with a sensitivity of 67.0% and a specificity of 77.8%. Some physicians may prefer conservative therapies without TKIs, or a watchful waiting strategy, in CCRCC patients with only small lung metastatic lesions [11]. Furthermore, cytokine therapies are still employed in CCRCC patients, especially in Japan, because of their low toxicity and ability to achieve long-term stable disease [12]. However, the present results suggest that smaller lung lesions are associated with a greater chance of response to TKIs, and it is therefore important not to miss the opportunity for early initiation of TKI treatment in patients with PD during watchful waiting periods or cytokine therapy.

Several studies have investigated the response of primary kidney lesions to TKIs [13-15]. Kroon et al. reported that smaller primary lesions were more responsive to treatment, and that tumors of 5–7 cm may benefit from neoadjuvant treatment followed by nephron-sparing surgery. In contrast, our results showed that the response of kidney lesions to sunitinib was independent of initial tumor size, and many smaller lesions exhibited no response. A possible explanation for this difference may be the selection of patients; most of the kidney lesions were investigated in the neoadjuvant setting in Kroon et al.’s study, while all the patients with kidney lesions in the current study had an extensive metastatic tumor burden. The different patient backgrounds may have led to different responses to TKIs, particularly in small kidney lesions.

CRP is an acute phase protein produced by the liver in response to various conditions, such as inflammation, infection, and malignancy [16]. In the cytokine era, elevated serum CRP level has been suggested as a biomarker for predicting poor survival in RCC patients [17-19]. Yasuda et al. recently demonstrated that CRP was a significant predictive marker for prognosis in metastatic RCC patients treated with TKIs [20]. In the current study, the size reduction of lung lesions in patients with high serum CRP levels was lower than that in patients with low CRP levels, irrespective of the initial size. This lower response to sunitinib in patients with higher serum CRP levels may be attributed to an aggressive disease status, reflected by higher CRP levels, the acquisition of resistance to therapeutic agents through an increase in inflammatory mediators in the cancer-cell microenvironment, or compromised drug metabolism induced by such mediators associated with CRP [21].

Tumor response to treatment is currently assessed by imaging based on RECIST criteria [22]. However, although marked central necrosis is often detected in lesions with a small size reduction after treatment with TKIs, RECIST only considers one-dimensional lesional size changes, suggesting that it may substantially underestimate the actual tumor response. Several studies recently reported novel criteria, which may improve response assessment by evaluating changes in tumor attenuation and morphology on contrast-enhanced computed tomography scans in addition to size changes [5,22-26]. The results of this study therefore need to be interpreted carefully, because lesions in different organs may exhibit distinct response patterns in imaging. Moreover, the current study did not demonstrate an association between tumor response and patient survival, and it is possible that percent change in tumor size might not correlate directly with survival. Further studies are needed to determine the influence of organ-specific response patterns to TKI treatment on survival.

## Conclusions

The results suggest that tumor-size reduction depends on initial tumor size and the organs involved, as well as systemic reaction to the lung tumor, as indicated by CRP levels. CCRCC patients with lung metastatic lesions < 20 mm in diameter and lower CRP levels may achieve greater reductions in tumor size with sunitinib therapy than those with extra-pulmonary lesions, lung lesions ≥ 20 mm in diameter, and/or higher CRP levels.

## Abbreviations

RCC: Renal cell carcinoma; TKI: Tyrosine kinase inhibitor; RECIST: Response evaluation criteria in solid tumors; CCRCC: Clear cell RCC; CTC-AE: Common Terminology criteria for Adverse Events; ROC: Receiver-operator curve; AUC: Area under the curve; CRP: C-reactive protein.

## Competing interests

Norihiko Tsuchiya and Tomonori Habuchi received honoraria from Pfizer Japan Inc.

## Authors’ contributions

NT, TY, JY, and TH were involved in the conception and design of the study. TY, KN, MS, and SM were involved in the provision of patients’ clinical data. NT and TH drafted the manuscript. SN, TI, SS supported the manuscript writing. All authors have read and approved the final manuscript.

## Pre-publication history

The pre-publication history for this paper can be accessed here:

http://www.biomedcentral.com/1471-2490/14/26/prepub
